# Could Novel Spinal Braces with Flexibility, Robotic Components, and Individualized Design Generate Sufficient Biomechanical Treatment Efficacy in Patients with Scoliosis?

**DOI:** 10.3390/bioengineering12101083

**Published:** 2025-10-05

**Authors:** Chen He, Jinkun Xie, Rong Pang, Bingshan Hu, Christina Zong-Hao Ma

**Affiliations:** 1 School of Health Science and Engineering, University of Shanghai for Science and Technology, Shanghai 200093, China; hechen@usst.edu.cn (C.H.); 18567285086@163.com (J.X.); pangrong23@163.com (R.P.); hbs922@163.com (B.H.); 2 Department of Biomedical Engineering, The Hong Kong Polytechnic University, Hong Kong SAR 999077, China; 3 Research Institute for Smart Ageing, The Hong Kong Polytechnic University, Hong Kong SAR 999077, China

**Keywords:** scoliosis, spinal brace, smart brace, spinal orthosis, biomechanical effect, treatment efficacy

## Abstract

Background: Patients with adolescent idiopathic scoliosis (AIS) require effective bracing to control curve progression. However, most traditional spinal braces commonly pose challenges in terms of undesired bulkiness and restricted mobility. Recent advancements have focused on innovative brace designs, utilizing novel materials and structural configurations to improve wearability and functionality. However, it remains unclear how effective these next-generation braces are biomechanically compared to traditional braces. Objectives: This review aimed to analyze the design features of next-generation AIS braces and assess their biomechanical effectiveness via reviewing contemporary studies. Methods: Studies on newly designed scoliosis braces over the past decade were searched in databases, including Web of Science, PubMed, ScienceDirect, Wiley, EBCOHost and SpringerLink. The Joanna Briggs Institute Critical Appraisal Checklist for Cohort Studies was adopted to evaluate the quality of the included studies. The data extracted for biomechanical effect analysis included brace components/materials, design principle, interfacial pressure, morphological changes, and intercomparison parameters. Results: A total of 19 studies encompassing 12 different kinds of braces met the inclusion/exclusion criteria. Clinical effectiveness was reported in 14 studies, with an average short-term Cobb angle correction of 25.4% (range: 12.41–34.3%) and long-term correction of 18.22% (range: 15.79–19.3%). This result aligned broadly with the previously reported efficacy of the traditional braces in short-term cases (range: 12.36–31.33%), but was lower than the long-term ones (range: 23.02–33.6%). Two included studies reported an interface pressure range between 6.0 kPa and 24.4 kPa for novel braces, which was comparable to that of the traditional braces (4.8–30.0 kPa). Additionally, five of six studies reported the trunk asymmetric parameters and demonstrated improvement in trunk alignment. Conclusions: This study demonstrates that most newly designed scoliosis braces could achieve comparable biomechanical efficacy to the conventional designs, particularly in interface pressure management and Cobb angle correction. However, future clinical adoption of these novel braces requires further improvements of ergonomic design and three-dimensional correction, as well as more investigation and rigorous evidence on the long-term treatment outcomes and cost-effectiveness.

## 1. Introduction

Scoliosis is a three-dimensional (3D) spinal deformity that primarily occurred in adolescents who are in the process of rapid growth [1,2]. Severe scoliosis may lead to some musculoskeletal, respiratory, and neurological problems [3,4]. Among all the treatment options, the spinal brace (or orthosis) intervention has been regarded as an effective conservative treatment for scoliosis, especially for the moderate curvatures [5,6].

Scoliosis braces, such as Milwaukee brace [7], Boston brace [8], Cheneau brace [9], Lyon brace [10], and Wilmington brace [11], have been used clinically for decades. These braces applied the three-point pressure principle: a correction force at the level of apical vertebrae and the counterforce at the upper end and lower end level of the scoliotic curve, via some correction pads and the shell of spinal braces [12]. These spinal braces have been proven effective in treating scoliosis, with over 18 h of wearing time every day [13]. The night-time braces, such as Charleston brace [14] and Providence brace [15], were developed for patients with mild scoliosis to wear only at night. However, these rigid spinal braces have the limitations of restraining trunk motion and producing discomfort during wearing, leading to poor compliance rate in patients with scoliosis [16].

To improve the compliance rate, some flexible braces, such as SpineCor brace [17] and TriaC brace [18], were developed in the 2000s to facilitate trunk movement and improve patients’ wearing comfort. Some previous studies have also reported higher compliance of the flexible braces than that of the rigid ones [19,20]. Nevertheless, a critical trade-off has emerged, as the corrective efficacy of these flexible braces has remained controversial and often less predictable [17].

In recent years, some novel scoliosis braces with new materials, structures, or functionality have been developed. These braces combined the elastic and rigid materials and were reported to be capable of correcting spinal deformity while allowing body movement [21]. Some more complexed electronic scoliosis robots, which were composed of three corrective platforms aligned with three-point pressure planes, were also developed to correct spinal curvature in three levels independently [22,23]. Proponents suggested that this design allowed the trunk to be maintained in a corrected position, potentially achieving the necessary correction while addressing the mobility limitations [24].

The developments of novel scoliosis brace imply potential effects in key biomechanical parameters—interface pressure distribution and morphological correction—compared to traditional braces. These parameters are critical, as interface pressure directly influences brace effectiveness and comfort, while morphological correction, as measured by Cobb angle reduction and body symmetry, reflects clinical success. Regarding interface pressure, previous studies reported that the overall interface pressure of the traditional braces ranged from 4.80 to 30.00 kPa, including the Boston brace (4.80–30.00 kPa) [25,26], Chêneau brace (7.34–9.45 kPa) [27], Milwaukee brace (7.53–9.31 kPa) [28,29], TLSO brace (7.09 kPa) [30], and Charleston brace (8.93–18.53 kPa) [31]. For morphological outcomes, evidence demonstrated that traditional braces achieved short-term (<6 months) Cobb angle reductions of 12.36% to 31.33%, across the SpineCor brace (17.36–21.35%) [32,33], Cheneau brace (15.50–31.33%) [34,35], Boston brace (19.4%) [34], and TLSO brace (12.36–15.90%) [32,33]. Meanwhile, the long-term (>6 months) treatment outcomes showed a further enhanced efficacy of 23.02–33.6%, based on the results of the SpineCor brace (31.34%) [36], Cheneau brace (25.6–30.89%) [34,35,37], and Boston brace (23.02–33.6%) [34,36,37]. Beyond the curvature correction, the patient’s body symmetry [38] would also be crucial, as it reflects trunk balance and alignment, which are vital for achieving effective scoliosis correction [39,40]. Although the interface pressure ranges and the short/long-term morphological outcomes were documented for traditional braces, there is an absence of evidence evaluating whether the novel brace designs could also achieve comparable biomechanical effectiveness or not, in terms of the pressure distribution, Cobb correction, and body symmetry parameters.

Most published review papers and meta-analysis studies have predominantly focused on synthesizing the Cobb angle progression prevention/correction of traditional brace [41,42,43], or comparing different traditional braces [44]. Some reviews have summarized the general ranges of the measured interface pressure across various traditional braces [45,46]. Some other reviews have focused on the impact of patient compliance or quality of life on the brace treatment efficacy [47,48]. However, there is a notable lack of reviews analyzing the innovative design features of more novel braces and critically evaluating the emerging evidence regarding their biomechanical performance and underlying treatment mechanisms. With the potential application of novel and smart scoliosis braces, it is necessary to systematically conduct a review and synthesis of the latest evidence on the biomechanical effect and clinical efficacy of the novel scoliosis braces, to guide future clinical practice and research directions.

To address the above-mentioned issues, the objectives of this review are (1) to identify the novel design features and trends of the reported novel scoliosis braces; and (2) to evaluate the evidence concerning the novel scoliosis braces’ biomechanical effects on interface pressure and morphological changes. It is expected that the findings of this study can offer synthesized evidence regarding the implementation of the newly developed scoliosis braces in clinical practice and inspire future brace designs for patients with scoliosis.

## 2. Materials and Methods

### 2.1. Search Strategy

Published studies were searched using a three-step search strategy. An initial search at MeSH browser was first undertaken to analyze the keywords contained in the titles and abstracts, and the index terms used to describe the article. The keywords that were identified for literature search were “AIS”, “scoliosis”, “spine deformity”, “brace/braces”, “orthotic device”, “orthosis/orthoses”, “biomechanical effect”, and “biomechanics”. A second search across databases of Web of Science, PubMed, ScienceDirect, Wiley, EBCOHost and SpringerLink was undertaken using all identified keywords and index terms. An example of the literature search results from Web of Science is provided in Table 1. Thirdly, the reference lists of the identified reports and studies were searched for additional studies.

The inclusion criteria were studies that (1) focused on a novel scoliosis brace; (2) provided a clear description of the brace’s design and components; (3) reported quantitative biomechanical outcomes; and (4) were published in English between 2014 and 2024. The exclusion criteria were studies that (1) focused on traditional scoliosis brace or minor modifications of traditional designs; (2) focused on spinal brace for non-scoliotic conditions; and (3) lacked essential design details or biomechanical data. Scoliosis braces were identified as traditional or novel based on their fundamental design philosophy and date of conception. Traditional braces were defined as clinically established, widely adopted designs validated through decades of use—including but not limited to the Milwaukee, Boston, Chêneau, Lyon, and SpineCor braces. Novel braces, by contrast, were developed and documented within the past decade (2014–2024) and incorporated substantial innovations in one or more domains: materials (use of non-traditional materials beyond conventional polyethylene plastics or elastic straps), structure (introduction of new structural concepts), or functionality (integration of novel capabilities). It is important to note that minor iterative adjustments or customizations of conventional braces did not qualify as a novel brace.

All identified studies were collated and uploaded into EndNote for removal of duplicate articles. Titles and abstracts were screened by two independent reviewers (J.X. and R.P.). The full text of the screened studies was assessed in detail to determine the compliance with the inclusion criteria by two independent reviewers (J.X. and R.P.). Exclusion of full-text sources that did not meet the inclusion criteria was documented along with the specific reasons. Any disagreements that arose between the two reviewers at each stage of the screening process were resolved through discussion, or with an additional reviewer (C.H.). This literature review was conducted and reported in accordance with the Preferred Reporting Items for Systematic Reviews and Meta-Analyses (PRISMA) guidelines and was registered with PROSPERO (Registration ID: CRD420251130681).

### 2.2. Assessment of Methodological Quality

Studies selected for retrieval were assessed by two independent reviewers for quality evaluation (J.X. and R.P.). The Joanna Briggs Institute (JBI) Critical Appraisal Checklist for Cohort Studies was adopted to assess the quality of the included studies. Any disagreements that arose between reviewers were resolved through discussion, or with a third reviewer (C.H.).

### 2.3. Extraction and Analysis

Data were extracted using a self-developed data extraction tool. The extracted data included brace components/materials, interface pressure, design principle, morphological changes, and intercomparison parameters. The draft data extraction tool was modified and revised as necessary during the process of extracting data from each included study.

## 3. Results

### 3.1. Search Results

**Figure 1:**
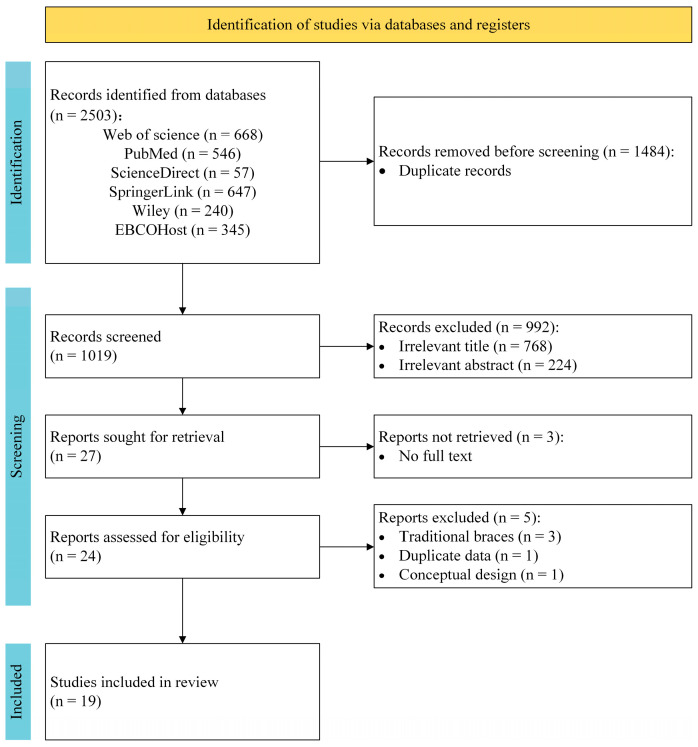
Prisma flow diagram.

Full details of the search results are illustrated in a PRISMA flow diagram (Figure 1). A systematic search across the above-reported databases yielded 2503 records initially. After removing 1484 duplicates, 1019 records were further screened for title/abstract, with 992 additional studies excluded. Full-text retrieval was then sought for the remaining 27 articles, with 3 articles inaccessible. The remaining 24 articles were assessed in full text, resulting in 5 exclusions. Finally, a total of 19 studies were included in this literature review.

### 3.2. Methodological Quality

The methodological quality of the included studies is presented in Table 2. Twelve articles were scored 5 points or above. Two articles were scored below 5 points, primarily attributed to the lack of control group, absence of follow-up or insufficient follow-up duration. Five articles were scored 0 points, because they were not clinical trials and thus did not meet the checklist criteria.

While the five articles that were scored 0 points were excluded from the subsequent biomechanical analysis due to the lack of clinical data/outcomes, these five articles were retained for inclusion in the novel design analysis section, as these studies provided essential, detailed descriptions of the novel features and concepts under investigation. Such information offered insights into the engineering rationale and technical specifications that cannot be derived solely from most clinical cohort studies. Furthermore, these conceptual and design-focused studies contributed to the understanding of the developmental history/trajectory of these novel technologies, facilitating a comprehensive review and synthesis of the brace design innovations.

### 3.3. Characteristics of the Included Studies

The brace components, correction principles, and experimental data extracted in each article are tabulated (Table 3), in which data from different articles of the same brace were merged together. Two studies [61,62] did not specify the names of their newly developed braces, which was designated as a Soft Brace (1st/2nd edition) in this review.

### 3.4. Novel Designs Features

**Figure 2:**
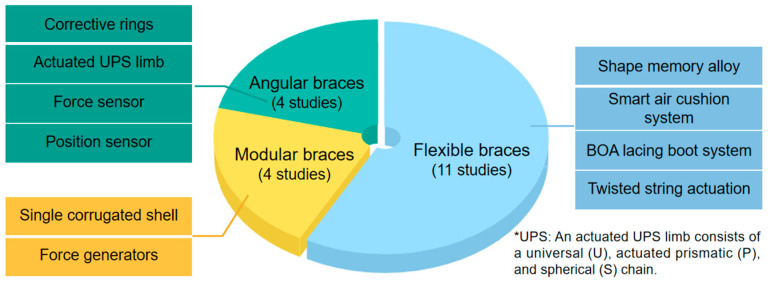
Novel design features of scoliosis braces.

The novel braces identified from 19 included studies could be categorized as flexible garment-style braces [5,49,50,51,52,53,54,55,56,57,58], angular braces [23,61,62,63], and modular braces [3,21,59,60] according to the structural design. The innovative features of novel braces are presented in Figure 2

Flexible garment-style braces included six braces named Posture Correction Girdle (1st–3rd editions), Anisotropic Textile Brace, Textile-based Scoliosis Brace, and Soft Active Dynamic Brace. Braces with flexibility emphasized flexible characteristics in materials or structures, aiming to reduce movement restrictions and improve comfort. These designs featured full-coverage vests enveloping patients’ shoulders, torso, and hips with flexible components. Angular braces included three braces named Soft Brace (1st–2nd edition) and RoSE Brace. These devices shared a common structure—three parallel rings positioned at the thoracic, lumbar, and pelvic regions, respectively—to provide spinal support and correction forces. Some angular braces incorporated robotic components of perceivable or controllable functional units, including but not limited to active or semi-active mechanical adjustment, sensing, monitoring, or automatic regulation devices, in the brace design.

Modular braces included two braces named FLEXpine and DSB (2nd edition). These braces covered the specific trunk segments with adjustable components to apply force. Such individualized designs can adapt according to changes in the body shape, curvature, or growth dynamics through modular, segment-replaceable, or adjustable elements.

For brace components, paddings were applied in four braces (i.e., Posture Correction Girdle [1st–2nd editions], Anisotropic Textile Brace, and Textile-based Scoliosis Brace) [5,49,50,51,52,53,54,56,57,58]. Some smart control systems, such as the smart airbag system, twisted string actuation (TSA), and actuated UPS (Universal joint, actuated Prismatic chain, Spherical joint) limb and sensor, were implemented in three braces (i.e., Posture Correction Girdle [3rd edition], Soft Active Dynamic Brace, and RoSE) [3,23,55,63]. Novel materials of shape memory alloy and SMA were applied in the Posture Correction Girdle (2nd edition) [50].

### 3.5. Analysis of Correction Principles

Nine studies mentioned the three-point pressure principle in their brace designs [3,5,21,55,56,57,58,59,60], whereas four studies introduced the displacement control as a methodological approach for achieving the three-dimensional spinal correction [23,61,62,63]. In addition to coronal correction, the Soft Active Dynamic Brace claimed an anti-rotational mechanism in the brace design [3], and the Posture Correction Girdle (3rd edition) reported an anti-rotational efficacy (shoulder–pelvic rotation decreased 56.6%) in their study [55].

### 3.6. Interface Pressure Measurement

Two studies quantified the interface pressure for novel braces [5,58]. Anisotropic Textile Brace demonstrated higher pressure range in thoracic region (6.0–24.4 kPa) than that of the lumbar region (6.1–9.7 kPa) [58]. Textile-based Scoliosis Brace exhibited a narrower pressure range of 17.62–20.98 kPa [5] in comparison with that of Charleston night-time braces (8.93–18.53 kPa) [31]. The overall pressure range of both thoracic and lumbar region reached 6.0–24.4 kPa.

### 3.7. Outcomes of Morphological Evaluation

Fourteen studies reported morphological changes of the spine and trunk upon or after wearing the brace, among which ten studies [21,50,52,53,54,56,57,58,59,60] reported the Cobb angle change, with a short-term correction averaging 25.4% (12.41–34.3%) in six studies and a long-term correction averaging 18.22% (15.79–19.3%) in three studies. Data from Liu et al. [50] (reporting an increase in Cobb angle after wearing brace) was excluded from the pooled analysis, due to the inconsistent tools adopted for curvature assessment before and after using the brace (radiograph versus motion capture assessment) in the study. The intercomparison among different braces showed that the Posture Correction Girdle (1st edition) demonstrated marginally inferior Cobb angle correction as compared to the SpineCor brace (16% vs. 21.3%) [33,52], and the Dynamic Spinal Brace (2nd edition) exhibited a comparable efficacy to TLSO in Cobb angle improvement (immediate: 34.3% vs. 38.1%; final: 19.3% vs. 22.65%) [60,65].

Beyond Cobb angle correction, the trunk asymmetry parameters were reported in six studies, including the reduced shoulder height difference from 1.27 to 0.16 cm [49], reduced shoulder tilt from 2.91° to 2.15° [50] and from 0.89° to 0.17° [55] in two sides, reduced pelvic tilt angle from 1.14° to 0.72° [55], changed sagittal vertical axis (SVA) angle from −21.2° to −26° [57], changed posterior trunk asymmetry index (POTSI) from 22.1 to 38.6% to 14.1–43.2% [58], and increased spinal pelvic obliquity (SPO) from 9.4° to 10.6° [51,55,59]. Additionally, the rotation correction was reported in two studies, including reduced shoulder–pelvic rotation from 1.22° to 0.53° [55] and reduced rotation angle in horizontal plane from 2.78° to 1.16° [51].

The duration of brace intervention varied considerably across different studies, ranging from 2 h [5,53,54,55,56,57,58] and 3 h [49] to 3 months [21,50] and 6 months [51,52,59,60]. The relevant assessments were primarily short-term, with eight studies evaluating the effects within 24 h and two studies conducting evaluations exceeding 1 year.

The participant recruitment also differed: three studies enrolled larger cohorts (with 18 [21], 52 [59], and 219 [60] participants), while the remaining twelve studies included smaller groups (5 ± 3 participants). Control groups were recruited in three studies [21,54,57].

## 4. Discussion

The potential application of novel scoliosis braces requires a systematic synthesis of current evidence concerning their biomechanical performance and therapeutic efficacy. This review aims to characterize emerging design innovations and evaluate the biomechanical evidence base. The synthesized findings demonstrates that the majority of recently developed brace designs achieve biomechanical performance comparable to conventional braces, particularly in managing interface pressure distributions and reducing Cobb angle magnitude.

### 4.1. Novel Designs

There are generally three different novel brace designs, including the flexible garment-based braces, angular braces, and modular braces. Each design has its pros and conc that need to be considered when selecting the appropriate prescription for patients with scoliosis. Further optimization is also needed for each of these novel brace designs. More details can be found below.

By integrating the elastic correction components within the fabric structure to deliver corrective forces, the flexible garment-based braces offered advantages in improving comfort, esthetics, and concealability [66]. This led to improved patient compliance, which was a critical factor in achieving sustained biomechanical efficacy [67,68]. Some flexible braces incorporated specialized material compositions and functional elements, to ensure consistent correction. For instance, the use of SMA has replaced the conventional resin-based support structures, to enhance durability and elasticity. Some more advanced functional modules, such as the Smart Air Cushion System, BOA Lacing Boot System and TSA, were employed to improve the adjustability and enable intelligent control of correction. However, the inherent flexibility of these braces may provide less restriction as compared to rigid braces [5,49,50,51,52,53,54,55,56,57,58]. Consequently, the successful correction relied partly on the patient’s active participation and conscious effort towards maintaining a proper posture [32].

Angular braces adopted an innovative design paradigm by utilizing three parallel rings (thoracic, lumbar, pelvic) to deliver targeted, adjustable three-dimensional forces [69]. The robotic Spine Exoskeleton (RoSE) integrated an actuated UPS limb as a programmable force generator, complemented by force and position sensors. The sensor array enabled real-time closed-loop monitoring and adjustment of both correction force vectors and patient trunk displacement within the brace. The independent control of each correction ring demonstrated enhanced capacity to address complex three-dimensional spinal deformities. However, the substantial weight and bulk might compromise patient compliance as compared to the conventional braces [70]. This dichotomy underscored the need for continued innovation to balance precise biomechanical control with the acceptable ergonomic practicality in spinal correction technologies. In contrast, the Soft Brace (2nd edition) utilized a corrugated geometric configuration to achieve multi-directional compliance. Concurrently, its force generators maintained a constant correction force (40–77.5 N) within a predefined displacement range based on the zero-stiffness principle [62]. This design optimized correction efficiency through force–displacement coupling.

Modular braces usually combined individual segments with elastic materials in the brace structure, ensuring effective correction of spinal deformities while minimizing the coverage of patient’s torso. The Soft Active Dynamic Brace exemplified this principle through four adjustable elastic bands controlled by lightweight twisted string actuators (TSAs), allowing for precise force modulation [3]. The DSB (2nd edition) adopted segmented architecture to accommodate diverse body shapes and discrete contact points. While this design exhibited insufficient pelvic support compared to the rigid brace incorporating rigid pelvic bases, further improvement was required on reinforcing the pelvic structure to enhance stability. The FLEXpine brace utilized a modular structure comprising curved, interlocking segments contoured to the torso. This configuration allowed localized adjustments and adaptation to spinal curvature changes over time. Nevertheless, its reliance on elastic components probably compromised the sustained pressure delivery. Integration of more rigid elements could be prioritized to improve pressure maintenance without sacrificing adaptability in future developments [21].

To sum up, the flexible brace systems prioritized comfort and compliance, angular designs maximized correction precision, and modular architectures leveraged the manufacturing advancements to balance adaptability and longevity. Future work could explore synergies between these different approaches, such as integrating sensor-driven feedback into modular frameworks or enhancing angular systems with flexible interfaces, to optimize the treatment outcomes in patients with scoliosis.

### 4.2. Correction Principles

Two fundamental correction mechanisms, including the force-controlled approach and displacement-controlled approach, were mentioned in the novel scoliosis braces. The force-controlled approach extended the traditional three-point force system (i.e., a primary convex force with two counterforces) in the coronal plane, by incorporating multi-planar force vectors, as seen in the Soft Active Dynamic Brace and the Posture Correction Girdle [3,49]. In contrast, the ring-shaped braces employed a displacement-controlled correction approach, where three corrective rings were positioned at different anatomical levels (corresponding to the three force application points in traditional three-point systems) to deliver correction force through electrodynamic actuators [63]. While optimal three-dimensional biomechanical correction was achievable from a mechanical perspective, the more complex interactions with the human torso’s musculoskeletal system rendered its practical application significantly [71]. Further efforts shall be made to improve the appearance, ergonomics, and application potential of such robotic designs, as well as the patient’s compliance while maintaining the biomechanical correction.

### 4.3. Interface Pressure

The interface pressure between the brace and the trunk served as a critical parameter for evaluating the biomechanical effects of spinal braces. The overall interface pressure range of novel braces (6.0–24.4 kPa) has been on par with that of traditional rigid braces (4.80–30.00 kPa) [25,26,27,28,29,30,31]. Specifically, the Anisotropic Textile Brace demonstrated a pressure range that closely aligned with traditional rigid braces [26,30], supporting the textile-based flexible designs as viable alternatives in interface pressure management in the future. Lower interface pressure was observed in the lumbar region than that of thoracic region, which likely stemmed from the anatomical and material factors. This is because the pliable soft tissues of the lumbar region, combined with the reduced correction capacity of flexible brace components in this area, may limit the force transmission efficiency [50,55,66]. Similarly, a Textile-based Scoliosis Brace demonstrated a narrower and higher range of interface pressure as compared to that of the Charleston night-time braces [27,31,64], indicating improved interface pressure consistency. This may have resulted from the replacement of elastic textile materials or the design of resin bones/hinged backbone, enabling targeted force application while reducing variability.

Notably, higher interface pressure did not necessarily translate to better correction outcomes. The correlation between interface pressure and Cobb angle reduction becomes non-linear beyond some specific force thresholds, suggesting diminished returns on spinal correction with uncontrolled pressure escalation [55,68]. Therefore, the focus in future clinical practice should be on achieving the optimal curve correction while maximizing the interface pressure within an appropriate range.

### 4.4. Morphological Evaluation

This review demonstrated that novel spinal braces achieved overall comparable Cobb angle correction to the traditional braces. The pooled short-term correction of 25.4% and long-term correction of 18.22% aligned with the expected range of brace management and broadly matched the reported traditional brace efficacy in short-term treatments (12.36% to 31.33%) [32,33,34,35]. However, the long-term correction efficacy is lower than in the traditional braces (23.02–33.6%) [34,35,36,37]. The inter-study comparisons also supported this finding. Specifically, two included studies reported that the Posture Correction Girdle was slightly less effective than SpineCor, and the DSB (2nd edition) performed equivalently to TLSO in both immediate and final correction [60,65]. The observed decrease in correction magnitude from short-term to long-term, alongside the reports of diminishing garment-style brace efficacy over time [5,60], suggests a potential challenge for achieving sustained effectiveness. This attenuation might be attributable to the fabric degradation in Soft Braces, compromising the elasticity, structural support, and correction forces that are necessary for long-term maintenance of correction. Meanwhile, the effectiveness of angular braces in correcting Cobb angles lacked clinical evidence, primarily due to the fabrication complexities and the relatively new emergence, making the long-term assessment challenging. Establishing these braces’ value required further study of underlying mechanisms, conducting iterative clinical trials, and tracking the Cobb angle changes to assess the short-term and long-term effectiveness.

The three-dimensional trunk asymmetric parameters showed a generally positive trend in improving the appearance of patients with scoliosis upon wearing the developed braces with novel designs. The reductions that occurred in shoulder height and tilt and pelvic tilt indicated improved coronal trunk alignment and symmetry [49,55]. The rotational deformities, including shoulder–pelvic rotation [55] and horizontal plane rotation [51], also showed substantial correction upon wearing the brace. The changes in SVA suggested a beneficial shift in sagittal alignment [57], though the response in POTSI varied across different individuals [58]. Overall, these results indicated the brace’s efficacy in reducing the shoulder imbalance, pelvic tilt, and rotational deformities of patients with scoliosis.

Although the reviewed braces with novel designs generally demonstrated biomechanical efficacy comparable to traditional braces, it shall be noted that limited previous studies have evaluated the cost-effectiveness of these novel braces. The braces incorporating advanced components (e.g., sensors, actuators) tended to have higher costs, which may not be an affordable option and may lead to limited access for patients, especially in resource-constrained settings. Future research should extend beyond the existing biomechanical validation to evaluate the cost-effectiveness of novel scoliosis braces, generating evidence for future clinical decision-making. For clinicians, this underscores the necessity of balancing treatment efficacy with affordability when making a clinical prescription, particularly in low- and middle-income countries. It shall be noted that when the treatment outcomes are similar, cost-efficient options should be prioritized over more expensive ones.

### 4.5. Limitations

This literature review has some limitations. Firstly, some included studies focused more on the design concept with less quantitative biomechanical evaluation of the scoliosis braces, making the synthesis of relevant clinical evidence unavailable in this review. Future studies need to move beyond the conceptual brace descriptions to conducting instrumented evaluations on patients, such as the radiographic analysis, pressure mapping, and 3D motion capture, to quantify the biomechanical effects. Secondly, the inhomogeneous data for evaluating the biomechanical effects of scoliosis brace in the included studies rendered it challenging to conduct a systematic comparison. It is critical to adopt standardized biomechanical parameters in future studies, to enable cross-study comparisons in future literature reviews/meta-analyses. Thirdly, while this review has adopted a systematic searching strategy to identify the qualified studies, papers not published in English were not included in this review. This may exclude some studies with the same focus.

## 5. Conclusions

This study demonstrates that most newly designed scoliosis braces could achieve comparable biomechanical efficacy to the conventional designs, particularly in interface pressure management and Cobb angle correction. However, future clinical adoption of these novel braces requires further improvements of ergonomic design and three-dimensional correction, as well as more investigation and rigorous evidence on the long-term treatment outcomes and cost-effectiveness.

## Figures and Tables

**Table 1:** Literature search from Web of Science.

Search	Query	Results
Web of science	Search:(((TS = (scoliosis)) OR TS = (Spine deformity)) OR TS = (AIS)) AND ((((((((TS = (orthosis*)) OR TS = (Orthotic Devices)) OR TS = (Orthosis)) OR TS = (Orthose*)) OR TS = (Parapodium*)) OR TS = (Conservative Treatment)) OR TS = (Conservative Management)) OR TS = (Conservative Therapy)) AND ((((((((((((((TS = (Biomechanical Phenomena)) OR TS = (Biomechanics)) OR TS = (Biomechanic*)) OR TS = (Biomechanic Phenomena)) OR TS = (Kinematics)) OR TS = (Invention*)) OR TS = (Technological Innovations)) OR TS = (design)) OR TS = (new)) OR TS = (devise*)) OR TS = (exploitation)) OR TS = (develop)) OR TS = (development)) OR TS = (exploiting))	668

Note: This search strategy employed the asterisk (*) as a truncation wildcard to retrieve plurals forms and variant endings (e.g., Orthosis* to capture Orthosis, Orthoses, Orthotic), thereby enhancing search sensitivity.

**Table 2:** Methodological quality of the included studies (n = 19).

No.	Author	1	2	3	4	5	6	7	8	9	10	11	Total
1	Liu et al. (2014) [49]	N	Y	Y	N	N	Y	Y	N	N	N	N	4/11
2	Liu et al. (2015) [50]	N	Y	Y	N	N	Y	Y	N	N	N	Y	5/11
3	Yip et al. (2016) [51]	N	Y	Y	N	N	Y	Y	Y	N	N	Y	6/11
4	Fok et al. (2018) [52]	N	N	Y	N	N	Y	Y	Y	N	N	Y	5/11
5	Liu et al. (2022) [53]	N	Y	Y	N	N	Y	Y	N	N	N	Y	5/11
6	Chan et al. (2018) [54]	Y	Y	Y	Y	Y	Y	Y	N	N	N	N	7/11
7	Ye et al. (2023) [55]	N	Y	Y	N	N	Y	Y	N	N	N	Y	5/11
8	Wang and Zing. (2017) [21]	N	Y	Y	Y	Y	Y	Y	Y	Y	Y	Y	10/11
9	Wong et al. (2020) [56]	N	Y	Y	N	N	Y	Y	N	N	N	Y	5/11
10	Wong et al. (2021) [57]	N	Y	Y	N	N	Y	Y	N	N	N	Y	5/11
11	Fok et al. (2022) [58]	N	N	Y	N	N	N	Y	N	N	N	Y	3/11
12	Fung et al. (2020) [5]	N	Y	Y	N	N	Y	Y	N	N	N	Y	5/11
13	Ali et al. (2021) [3]	N	N	N	N	N	N	N	N	N	N	N	0/11
14	Nakamura et al. (2014) [59]	N	Y	Y	N	N	Y	Y	Y	Y	Y	Y	8/11
15	Kajiura et al. (2019) [60]	N	Y	Y	N	N	Y	Y	Y	Y	N	Y	7/11
16	Ring and Kim. (2016) [61]	N	N	N	N	N	N	N	N	N	N	N	0/11
17	Nijssen et al. (2017) [62]	N	N	N	N	N	N	N	N	N	N	N	0/11
18	Park et al. (2015) [63]	N	N	N	N	N	N	N	N	N	N	N	0/11
19	Park et al. (2018) [23]	N	N	N	N	N	N	N	N	N	N	N	0/11

Y: Yes; N: No.

**Table 3:** Characteristics of the included studies.

NO.	Authors	Brace Design			Clinical Outcomes		
		Brace Name/Type	Brace Components	Correction Principle	Subjects	Study Duration	Results
1.	Liu et al. (2014) [49]	Posture correction girdle (1st edition)	Resin bones; Elastic straps; Pads.	Elastic stretch; Corrective point pressure force	N = 7; 11.43 ± 0.98 ys; Cobb = 10–20°	3 h	Shoulder height difference: 1.27 to 0.16 cm; Front bending: 30 to 15 cm; Lateral bending: 45 to 42.5 cm (Left), 45.5 to 39.5 cm (Right)
2.	Liu et al. (2015) [50]				N = 9; 11.33 ± 1.00 ys; Cobb = 12.33° ± 4.99°	8 h/d for 3 m	Cobb increases: 18.29° ± 3.82°; Shoulder tilt decrease: 2.91° ± 1.30° to 2.15° ± 0.91°
3.	Yip et al. (2016) [51]				N = 7; 11.14 ± 0.90 ys; Cobb = 9.43° ± 6.11° (T); 10.57° ± 4.79° (L)	8 h/d for 6 m	Rotation angle decrease: 2.78° to 1.16°
4.	Fok et al. (2018) [52]				N = 10; 13.5 ± 1.4 ys; Cobb = 19.0° ± 6.4°	8 h/d for 6 m	Cobb decrease: 16.0° ± 7.7° Less cobb correction than that of SpineCor brace [33]
5.	Liu et al. (2022) [53]				N = 4; 12.25 ± 0.50 ys; Cobb = 15.78° ± 2.63°	2 h	Cobb decrease: 11° ± 4.55°
6.	Chan et al. (2018) [54]	Posture correction girdle (2nd edition)	Shape Memory Alloy (SMA); Elastic straps; Semi-rigid Pads.	Elastic stretch; Corrective point pressure	Study Group (2nd edition): N = 1; 14 ys; Cobb = 34° (T)/24° (L); Control Group (1nd edition): N = 1; 14 ys; Cobb = 28.9°	2 h	Cobb angle decrease: Study Group: 22.7° (T)/23.8° (L); Control Group: 21.4°
7.	Ye et al. (2023) [55]	Posture correction girdle (3rd edition)	Resin bones; Elastic straps; Smart air cushion system	Dynamic pressure control via airbag; Three-point pressure	N = 3; 12.33 ± 0.47 ys; Cobb = 13.60° ± 3.45°	2 h	Shoulder tilt decreases 53.8% (average 0.89° to 0.17°); pelvic tilt decreases 36.8% (average 1.14° to 0.72°); shoulder–pelvic rotation decreases 56.6% (average 1.22° to 0.53°)
8.	Wang and Zing. (2017) [21]	FLEXpine	Soft frame; Elastic bands.	Three-point pressure;	Study Group: N = 10; wear FLEXpine or traditional brace; Cobb: 16.45° ± 0.98° Control Group: N = 8; wear FLEXpine brace With FLEXpine Exercise program; Cobb: 7.65° ± 2.5°	3 m	Cobb angle decrease: Study Group: 13.23° ± 3.24°; Control Group: 4.55° ± 2.32°
9.	Wong et al. (2020) [56]	Anisotropic textile brace	Elastic textile material; Hinged artificial backbone; Pads.	Three-point pressure	N = 1; 12 ys; Cobb = 21°	2 h	Cobb angle decrease: 15.4°
10.	Wong et al. (2021) [57]				N = 1; 11 ys; Cobb: 23.1° (T)/27.8° (L); Study Group (Boston); Control Group (Anisotropic textile brace)	2 h	Cobb angle decrease: Study Group: 14.6° (T)/25.2° (L); SVA (−21.2 to −8.2) Control Group: 14.8° (T)/22.6° (L) SVA (−21.2° to −26°)
11.	Fok et al. (2022) [58]				N = 5; 12.2 ± 0.45 ys; Cobb: 20.7° ± 4.13° (T) 20.2° ± 2.13° (L)	2 h	POTSI decrease: 22.1–38.6% to 14.1–43.2%; Cobb angle decrease: 11.9° ± 9.22° (T); 20.1° ± 5.39° (L) Interfacial pressure: 6.0–24.4 kPa(T)/6.1–9.7 kPa (L) (Comparable to rigid brace [26,30])
12.	Fung et al. (2020) [5]	Textile-based scoliosis brace	Pads; Rigid straps; BOA lancing system; Resin Bones.	Three-point pressure	N = 1; 21 ys; Cobb: 11.1° (T)/24.8° (L)	2 h	Interface pressure: 17.62–20.98 kPa (comparable to rigid brace [27,31,64])
13.	Ali et al. (2021) [3]	Soft Active Dynamic Brace	Twisted String Actuation (TSA); Corrective band.	Three-point pressure correction; Trunk rotation with lateral flexion	No subject	Not applicable	Not Applicable
14.	Nakamura et al. (2014) [59]	Dynamic Spinal Brace (DSB) (1st edition)	Rigid shell; Polycarbonate strut; Corrective band.	Posture control Three-point pressure	N = 52; 10 ys; Cobb: 41.9° ± 16.91° SPO: 9.4° ± 7.01°	20.8 m (6.8–35.7 m)	Cobb angle decrease: 36.7° ± 16.21° (1 h); 49.4° ± 25.4° (long term) SPO decrease: 7.4° ± 5.71° (1 h); 10.6° ± 9.01° (long term)
15.	Kajiura et al. (2019) [60]	Dynamic Spinal Brace (DSB) (2nd edition)	Elastic ring-shaped support	Posture control Three-point pressure	N = 219; 13.4 ys; Cobb ≥ 20°	6 y (3–9 y)	Cobb angle decrease: 34.3% (1 h); 19.3% (long term) Comparable cobb correction to TLSO [65]
16.	Ring and kim (2016) [61]	Soft brace (1st edition)	Corrective rings	Spine displacement control	No subject	Not applicable	Not applicable
17.	Nijssen et al. (2017) [62]	Soft brace (2nd edition)	Shell mechanisms; Force generators.	Two-fold force-controlled correction method	No subject	Not applicable	Not applicable
18.	Park et al. (2015) [63]	Robotic Spine Exoskeleton (RoSE)	Corrective rings; Stewart platforms; UPS configuration; Force and position sensor.	Displacement control; 3D dynamic adjustable correction	No subject	Not applicable	Not applicable
19.	Park et al. (2018) [23]				No subject	Not applicable	Not applicable

T—thoracic curve, L—lumbar curve, POTSI—posterior trunk asymmetry index, m—month, N—number, ys—years, SVA—sagittal vertical axis, SPO—spinal pelvic obliquity, UPS—universal prismatic and spherical joint. Notes: In this table, different colors represent distinct brace designs. Variations in shade within a single color represent different clinical evaluations or design stages of the same brace design.

## Data Availability

All data generated or analyzed in this study are included in this published article.

## Abbreviations

The following abbreviations are used in this manuscript:

AIS	Adolescent Idiopathic Scoliosis
ATSI	Anterior Trunk Symmetry Index
DSB	Dynamic Spinal Brace
L/T curve	Lumbar curve/Thoracic curve
m	month
No.	Number
POTSI	Posterior Trunk Asymmetry Index
PRISMA	The Preferred Reporting Items for Systematic Reviews and Meta-Analyses
SMA	Shape Memory Alloy
SPO	Spinal Pelvic Obliquity
SVA	Sagittal Vertical Axis
TLSO	Thoracic Lumbosacral Orthosis
TSA	Twisted String Actuation
UPS	The Universal Prismatic and Spherical joint
ys	years

## References

[1] Niu X., Yang C., Tian B., Li X., Zheng S., Cong D., Han J., Agrawal S.K. (2018). Investigation of robotic braces for patients with idiopathic scoliosis (IS)—Review of the literature and description of a novel brace. J. Mech. Med. Biol..

[2] Stokes I.A., Bigalow L.C., Moreland M.S. (1987). Three-dimensional spinal curvature in idiopathic scoliosis. J. Orthop. Res..

[3] Ali A., Fontanari V., Fontana M., Schmoelz W. Soft Active Dynamic Brace for Spinal Deformities. Proceedings of the 14th International Joint Conference on Biomedical Engineering Systems and Technologies.

[4] Weinstein S.L., Dolan L.A., Cheng J.C.Y., Danielsson A., Morcuende J.A. (2008). Adolescent idiopathic scoliosis. Lancet.

[5] Fung O.H.-Y., Yip J., Cheung M.-C., Yick K.-L., Kwan K.Y.-H., Cheung K.M.-C., Cheung J.P.-Y., Tse C.-Y. (2020). Exploring mass customization and textile application in medical products: Re-designing scoliosis brace for shorter production lead time and better quality of life. Text. Res. J..

[6] Negrini S., Aulisa A.G., Aulisa L., Circo A.B., de Mauroy J.C., Durmala J., Grivas T.B., Knott P., Kotwicki T., Maruyama T. (2012). 2011 SOSORT guidelines: Orthopaedic and Rehabilitation treatment of idiopathic scoliosis during growth. Scoliosis.

[7] Keim H.A. (1971). The Milwaukee brace for treatment of scoliosis. J. Pediatr..

[8] Watts H.G., Hall J.E., Stanish W. (1977). The Boston brace system for the treatment of low thoracic and lumbar scoliosis by the use of a girdle without superstructure. Clin. Orthop. Relat. Res..

[9] De Giorgi S., Piazzolla A., Tafuri S., Borracci C., Martucci A., De Giorgi G. (2013). Chneau brace for adolescent idiopathic scoliosis: Long-term results. Can it prevent surgery?. Eur. Spine J..

[10] de Mauroy J.C., Lecante C., Barral F., Daureu D., Gualerzi S., Gagliano R. (2008). The Lyon brace. Disabil. Rehabil. Assist. Technol..

[11] Bassett G.S., Bunnell W.P., MacEwen G.D. (1986). Treatment of idiopathic scoliosis with the Wilmington brace. Results in patients with a twenty to thirty-nine-degree curve. J. Bone Joint Surg. Am..

[12] Grivas T.B., Kaspiris A. (2010). European Braces Widely Used for Conservative Scoliosis Treatment.

[13] Weinstein S.L., Dolan L.A., Wright J.G., Dobbs M.B. (2013). Effects of bracing in adolescents with idiopathic scoliosis. N. Engl. J. Med..

[14] Lee C.S., Hwang C.J., Kim D.J., Kim J.H., Kim Y.T., Lee M.Y., Yoon S.J., Lee D.H. (2012). Effectiveness of the Charleston Night-time Bending Brace in the Treatment of Adolescent Idiopathic Scoliosis. J. Pediatr. Orthoped..

[15] Wiemann J.M., Shah S.A., Price C.T. (2014). Nighttime Bracing Versus Observation for Early Adolescent Idiopathic Scoliosis. J. Pediatr. Orthoped..

[16] Karol L.A., Virostek D., Felton K., Wheeler L. (2016). Effect of Compliance Counseling on Brace Use and Success in Patients with Adolescent Idiopathic Scoliosis. J. Bone Joint Surg. Am..

[17] Coillard C., Leroux M.A., Zabjek K.F., Rivard C.H. (2003). SpineCor--a non-rigid brace for the treatment of idiopathic scoliosis: Post-treatment results. Eur. Spine J..

[18] Veldhuizen A.G., Cheung J., Bulthuis G.J., Nijenbanning G. (2002). A new orthotic device in the non-operative treatment of idiopathic scoliosis. Med. Eng. Phys..

[19] Gonzalez Vicente L., Jimenez Barrios M., Gonzalez-Santos J., Santamaria-Pelaez M., Soto-Camara R., Mielgo-Ayuso J., Fernandez-Lazaro D., Gonzalez-Bernal J.J. (2021). The ISJ 3D Brace, a Providence Brace Evolution, as a Surgery Prevention Method in Idiopathic Scoliosis. J. Clin. Med..

[20] Capek V., Baranto A., Brisby H., Westin O. (2023). Nighttime versus Fulltime Brace Treatment for Adolescent Idiopathic Scoliosis: Which Brace to Choose? A Retrospective Study on 358 Patients. J. Clin. Med..

[21] Wang S.V., Zing S. (2017). Bracing Effects of the Flexpine in Scoliosis Patients. Am. Sci. Res. J. Eng. Technol. Sci..

[22] Niu X., Yang C., Han J., Agrawal S.K. (2018). Concept design of a novel robotic spinal brace for the treatment of scoliosis. Proc. Inst. Mech. Eng. Part H-J. Eng. Med..

[23] Park J.H., Stegall P.R., Roye D.P., Agrawal S.K. (2018). Robotic Spine Exoskeleton (RoSE): Characterizing the 3-D Stiffness of the Human Torso in the Treatment of Spine Deformity. IEEE Trans. Neural Syst. Rehabil. Eng..

[24] Ray R., Nouaille L., Colobert B., Calistri L., Poisson G. (2023). Design and position control of a robotic brace dedicated to the treatment of scoliosis. Robotica.

[25] Mac-Thiong J.M., Petit Y., Aubin C.E., Delorme S., Dansereau J., Labelle H. (2004). Biomechanical evaluation of the Boston brace system for the treatment of adolescent idiopathic scoliosis: Relationship between strap tension and brace interface forces. Spine.

[26] van den Hout J.A., van Rhijn L.W., van den Munckhof R.J., van Ooy A. (2002). Interface corrective force measurements in Boston brace treatment. Eur. Spine J..

[27] Pham V.M., Houilliez A., Schill A., Carpentier A., Herbaux B., Thevenon A. (2008). Study of the pressures applied by a Cheneau brace for correction of adolescent idiopathic scoliosis. Prosthet. Orthot. Int..

[28] Babaee T., Kamyab M., Ahmadi A., Sanjari M.A., Ganjavian M.S. (2017). Measurement of Milwaukee Brace Pad Pressure in Adolescent Round Back Deformity Treatment. Asian Spine J..

[29] Wong M.S., Evans J.H. (1998). Biomechanical evaluation of the Milwaukee brace. Prosthet. Orthot. Int..

[30] Wong M.S., Mak A.F., Luk K.D., Evans J.H., Brown B. (2000). Effectiveness and biomechanics of spinal orthoses in the treatment of adolescent idiopathic scoliosis (AIS). Prosthet. Orthot. Int..

[31] Lou E., Durdle N.G., Raso V.J., Hill D.L. (1994). A system for measuring pressures exerted by braces in the treatment of scoliosis. IEEE Trans. Instrum. Meas..

[32] Wong M.S., Cheng J.C.Y., Lam T.P., Ng B.K.W., Sin S.W., Lee-Shum S.L.F., Chow D.H.K., Tam S.Y.P. (2008). The effect of rigid versus flexible spinal orthosis on the clinical efficacy and acceptance of the patients with adolescent idiopathic scoliosis. Spine.

[33] Guo J., Lam T.P., Wong M.S., Ng B.K.W., Lee K.M., Liu K.L., Hung L.H., Lau A.H.Y., Sin S.W., Kwok W.K. (2014). A prospective randomized controlled study on the treatment outcome of SpineCor brace versus rigid brace for adolescent idiopathic scoliosis with follow-up according to the SRS standardized criteria. Eur. Spine J..

[34] Karimi M., Nadi A. (2024). Comparative analysis of Boston and Cheneau braces in treating scoliosis: A 2-year follow-up study on curve reduction. N. Am. Spine Soc. J..

[35] Korovessis P., Syrimpeis V., Tsekouras V., Vardakastanis K., Fennema P. (2018). Effect of the Cheneau Brace in the Natural History of Moderate Adolescent Idiopathic Scoliosis in Girls: Cohort Analysis of a Selected Homogenous Population of 100 Consecutive Skeletally Immature Patients. Spine Deform..

[36] Gutman G., Benoit M., Joncas J., Beauséjour M., Barchi S., Labelle H., Parent S., Mac-Thiong J.M. (2016). The effectiveness of the SpineCor brace for the conservative treatment of adolescent idiopathic scoliosis. Comparison with the Boston brace. Spine J..

[37] Minsk M.K., Venuti K.D., Daumit G.L., Sponseller P.D. (2017). Effectiveness of the Rigo Cheneau versus Boston-style orthoses for adolescent idiopathic scoliosis: A retrospective study. Scoliosis Spinal Disord..

[38] Zaina F., Negrini S., Atanasio S. (2009). TRACE (Trunk Aesthetic Clinical Evaluation), a routine clinical tool to evaluate aesthetics in scoliosis patients: Development from the Aesthetic Index (AI) and repeatability. Scoliosis.

[39] Han K.-S., Kim G.-W., Kang S.-R., Ko M.-H., Seo J.-H. (2020). Clinical evaluation of the effectiveness of a new orthotic device for the non-operative treatment of scoliosis. Technol. Health Care.

[40] Drummond D., Breed A.L., Narechania R. (1985). Relationship of spine deformity and pelvic obliquity on sitting pressure distributions and decubitus ulceration. J. Pediatr. Orthop..

[41] Ruffilli A., Fiore M., Barile F., Pasini S., Faldini C. (2021). Evaluation of night-time bracing efficacy in the treatment of adolescent idiopathic scoliosis: A systematic review. Spine Deform..

[42] Angelliaume A., Pfirrmann C., Alhada T., Sales de Gauzy J. (2025). Non-operative treatment of adolescent idiopathic scoliosis. Orthop. Traumatol. Surg. Res. OTSR.

[43] Karavidas N. (2019). Bracing In The Treatment Of Adolescent Idiopathic Scoliosis: Evidence To Date. Adolesc. Health Med. Ther..

[44] Lee Y.J., Wang W.J., Mohamad S.M., Chandren J.R., Abd Gani S.M., Chung W.H., Chiu C.K., Chan C.Y.W. (2024). A comparison between Boston brace and European braces in the treatment of adolescent idiopathic scoliosis (AIS) patients: A systematic review based on the standardised Scoliosis Research Society (SRS) inclusion criteria for brace treatment. Eur. Spine J..

[45] Luo C., Wu H., Liu W., Luo Y., Jie Y., Ma C.Z., Wong M. (2024). The Biomechanics of Spinal Orthoses for Adolescent Idiopathic Scoliosis: A Systematic Review of the Controlling Forces. Bioengineering.

[46] Nie W.Z., Ye M., Wang Z.Y. (2008). Infinite models in scoliosis: A review of the literature and analysis of personal experience. Biomed. Tech..

[47] Rahimi S., Kiaghadi A., Fallahian N. (2020). Effective factors on brace compliance in idiopathic scoliosis: A literature review. Disabil. Rehabil. Assist. Technol..

[48] Wang H., Tetteroo D., Arts J.J.C., Markopoulos P., Ito K. (2021). Quality of life of adolescent idiopathic scoliosis patients under brace treatment: A brief communication of literature review. Qual. Life Res..

[49] Liu P., Yip J., Yick K., Yuen C., Law D. (2014). An Ergonomic Flexible Girdle Design for Preteen and Teenage Girls with Early Scoliosis. J. Fiber Bioeng. Inform..

[50] Liu P.-Y., Yip J., Yick K.-L., Yuen C.-W., Tse C.-Y., Ng Z., Law D. (2015). Effects of a tailor-made girdle on posture of adolescents with early scoliosis. Text. Res. J..

[51] Yip J., Liu P.-Y., Yick K.-L., Cheung M.-C., Tse C.-Y., Ng Z. (2016). Effect of a Functional Garment on Postural Control for Adolescents with Early Scoliosis: A Six-Month Wear Trial Study. Advances in Physical Ergonomics and Human Factors, Proceedings of the AHFE 2016 International Conference on Physical Ergonomics and Human Factors, Orlando, FL, USA, 27–31 July 2016.

[52] Fok Q., Yip J., Yick K.L., Ng S.P., Tse C.Y. (2018). Effectiveness of Posture Correction Girdle as Conservative Treatment for Adolescent Idiopathic Scoliosis: A Preliminary Study. Orthop. Res. Online J..

[53] Liu P., Yip J., Chen B., He L., Cheung J., Yick K., Ng Z. (2022). Immediate Effects of Posture Correction Girdle on Adolescents with Early Scoliosis. Healthcare and Medical Devices.

[54] Chan W.Y., Yip J., Yick K.L., Ng S.P., Lu L., Cheung K.M., Kwan K.Y., Cheung J.P., Yeung K.W., Tse C.Y. (2018). Mechanical and Clinical Evaluation of a Shape Memory Alloy and Conventional Struts in a Flexible Scoliotic Brace. Ann. Biomed. Eng..

[55] Ye Z., Yip J., Cheung J.P.Y., Liang R., Zhang J., Li X., Tong R.K.-Y. (2023). Posture correction girdle with intelligent padding system to dynamically adjust the pressure distribution and correct the scoliotic spine. Healthcare and Medical Devices.

[56] Wong S.-H., Yip J., Yick K., Ng Z. Preliminary wear trial of anisotropic textile brace for adolescent idiopathic scoliosis. Proceedings of the ISERD International Conference.

[57] Wong C.S., Yip J., Yick K., Ng Z.S. A Case Study of Initial In-Brace Spinal Correction of Anisotropic Textile Brace and Boston Brace. Proceedings of the AHFE Virtual Conference on Human Factors and Ergonomics in Healthcare and Medical Devices, Electr Network.

[58] Fok Q., Yip J., Yick K., Ng S. (2022). Design and fabrication of anisotropic textile brace for exerting corrective forces on spinal curvature. J. Ind. Text..

[59] Nakamura N., Uesugi M., Inaba Y., Machida J., Okuzumi S., Saito T. (2014). Use of dynamic spinal brace in the management of neuromuscular scoliosis: A preliminary report. J. Pediatr. Orthop. B.

[60] Kajiura I., Kawabata H., Okawa A., Minobe Y., Matsuyama M., Yoshida K., Suzuki T. (2019). Concept and treatment outcomes of dynamic spinal brace for scoliosis in cerebral palsy. J. Pediatr. Orthop. B.

[61] Ring J.B., Kim C. A passive brace to improve activities of daily living utilizing compliant parallel mechanisms. Proceedings of the ASME International Design Engineering Technical Conferences and Computers and Information in Engineering Conference (IDETC/CIE).

[62] Nijssen J.P.A., Radaelli G., Herder J.L., Kim C.J., Ring J.B. Design and Analysis of a Shell Mechanism Based Two-Fold Force Controlled Scoliosis Brace. Proceedings of the ASME International Design Engineering Technical Conferences and Computers and Information in Engineering Conference.

[63] Park J.-H., Stegall P., Agrawal S.K. Dynamic Brace for Correction of Abnormal Postures of the Human Spine. Proceedings of the IEEE International Conference on Robotics and Automation (ICRA).

[64] Périé D., Aubin C.E., Petit Y., Beauséjour M., Dansereau J., Labelle H. (2003). Boston brace correction in idiopathic scoliosis: A biomechanical study. Spine.

[65] Terjesen T., Lange J.E., Steen H. (2000). Treatment of scoliosis with spinal bracing in quadriplegic cerebral palsy. Dev. Med. Child. Neurol..

[66] Hui C.-L., Piao J., Wong M.S., Chen Z. (2020). Study of Textile Fabric Materials used in Spinal Braces for Scoliosis. J. Med. Biol. Eng..

[67] Aulisa A.G., Giordano M., Falciglia F., Marzetti E., Poscia A., Guzzanti V. (2014). Correlation between compliance and brace treatment in juvenile and adolescent idiopathic scoliosis: SOSORT 2014 award winner. Scoliosis.

[68] Haleem S., Nnadi C. (2018). Scoliosis: A review. Paediatr. Child Health.

[69] Guo X., Zhou Z., Mai J., Wang Q. Kinematic and Kinetic Analysis of 3-RPR Based Robotic Lumbar Brace. Proceedings of the IEEE/ASME International Conference on Advanced Intelligent Mechatronics (AIM), Electr Network.

[70] Herget G.W., Patermann S., Strohm P.C., Zwingmann J., Eichelberger P., SÜDkamp N.P., HirschmÜLler A. (2017). Spinal Orthoses: The Crucial Role of Comfort on Compliance of Wearing—Monocentric Prospective Pilot Study of Randomized Cross-Over Design. Acta Chir. Orthop. et Traumatol. Cechoslov..

[71] Niu X., Yang C., Tian B., Li X., Han J. (2019). Modal Decoupled Dynamics Feed-Forward Active Force Control of Spatial Multi-DOF Parallel Robotic Manipulator. Math. Probl. Eng..

